# Time-resolved Raman spectroscopy using a CMOS SPAD array to remove fluorescent and fibre Raman backgrounds

**DOI:** 10.1364/BOE.560826

**Published:** 2025-06-17

**Authors:** Caitlin S. Tye, András Kufcsák, Calum A. Ross, Katjana Ehrlich, Robert K. Henderson, Michael G. Tanner

**Affiliations:** 1 Scottish Universities Physics Alliance (SUPA), Institute of Photonics and Quantum Sciences, Heriot-Watt University, Edinburgh, UK; 2 now at Department of Biomedical Engineering, University of California, Davis, California, USA; 3 Institute for Micro and Nano Systems, University of Edinburgh, Edinburgh, UK

## Abstract

Raman spectroscopy is limited by weak signals, fluorescence interference, and fibre-induced backgrounds. To address this, a 512-pixel complementary-metal-oxide semiconductor (CMOS) single-photon avalanche diode (SPAD) line sensor array with on-chip timing electronics was employed to enable the detection of weak Raman signals and use time gating to suppress background fluorescence and fibre-induced Raman signal. The functionality of this compact optical system is initially demonstrated in a free space setup, achieving an improved signal enhancement within shorter measurement times of 30 s. Finally, to support the development of miniaturised single fibre based Raman probes, the removal of fibre background is demonstrated through remote detection of paracetamol and other samples using time-gating and a 1 m 50 µm core multimode fibre.

## 1. Introduction

Raman spectroscopy uses the Raman spectra of a sample to analyse its chemical composition. Each Raman peak present in the Raman spectra of the sample corresponds to an interaction between the incident light and a vibrational mode of a molecule, subsequently enabling the identification of the molecules present. Due to the non-invasive nature of this technique, it is used in various fields such as pharmaceuticals [1], planetary science [2], and food agriculture [3,4]. In the medical field it is used to detect cancer [5–7], measure glucose levels [8,9] and detect different diseases [10,11]. Additionally, in-vivo Raman spectroscopy can be performed by using a fibre-optic Raman probe [12–15].

A limiting factor of Raman spectroscopy is the strength of the Raman signal compared to background signals. Certain samples, such as biological samples, have a stronger fluorescence signal than Raman signal which decreases the signal to noise ratio (SNR) [14]. Furthermore, in fibre-optic measurements, a large Raman signal can be generated within the optical fibre due to the extended interaction length, introducing additional background and noise to the overall measurement and masking the Raman signal from the sample.

To overcome the limitation of fluorescence, instrumental and computational approaches have been used to reduce or subtract the fluorescent signal from the sample. Polynomial fitting is a computational method used to reduce fluorescence signals in post-processing [16], however, this can lead to errors in mathematical estimation and lead to distortions of the Raman spectra [17,18] and shot noise is still present in the resulting spectra. Experimentally, photo-bleaching has been used to inhibit the fluorophores’ ability to fluoresce, however, this can result in irreversible damage to the molecule [19]. It is possible to use an excitation wavelength where the sample exhibits reduced fluorescence [20], however, not all fluorescence is removed, and wavelengths are often chosen for practical reasons of sources and detectors. An efficient method to remove fluorescence is to use multiple shifted excitations and apply a statistical model to compute the Raman signal, however, the removal of Raman generated by the fibre is still challenging and requires special probe designs [21]. Alternatively, time-domain approaches can be used to separate Raman and fluorescence as Raman scattering occurs instantaneously whereas fluorescence occurs on the pico-second to nano-second time scale [19]. Time-resolved methods can be achieved optically using a Kerr gate [22,23], time-gated detectors such as an intensified charge-coupled device camera [24,25], or photon counting detectors such as complementary metal-oxide-semiconductor (CMOS) single-photon avalanche diodes (SPADs) [26–28].

Kostamovaara et al. [17] show successful fluorescence suppression using time-gating techniques with a scanning spectrometer, however, this requires long measurement times of 240 s per spectral measurement point. Wang et al. [29] achieved shorter measurement times of 3 s per measurement with their scanning system, however, this still results in overall long measurement times of 1209 s for their spectral range. To further decrease overall measurement time, Usai et al. [30] and Finlayson et al. [31] used the same line sensor used in this work to simultaneously measure each spectral measurement point, resulting in measurement times of 2 min and 10 s, respectively. Their work demonstrates proof of concept of enhancement of Raman signals, where the few most significant Raman peaks are observed. We move beyond this to demonstrate Raman spectra with fine detail comparable to features observed in reference standard data, however, with increased visibility.

This time-resolved technique can also be extended to a fibre probe setup [32], enabling the separation of Raman scattering generated in the sample and the core of a standard multimode optical fibre. Currently, approaches used to mitigate fibre generated Raman signals include using shifted excitation Raman difference spectroscopy [18], however, shot noise from the subtracted fibre Raman background still adversely affects the resulting spectrum. Complex fibre probe designs separate the illumination and collection paths, removing most of the Raman background [33]. Alternatively, optics can be placed at the distal end of the fibre where dimensions of these optical components have been reduced to approximately 1 mm by using advanced manufacturing techniques [34]. The size of these optics determines the minimum diameter of the fibre-optic probe, which can be used for applications such as endoscopy. By using time-resolved techniques, further miniaturisation of low-cost probes is possible as a standard multimode fibre can be used, allowing the size of the optical fibre to be the limiting factor for the size of the probe rather than distal optics. It is believed this work is the first successful demonstration of this. Miniaturising fibre probes extends their medical applications from hollow organs to solid organs as the probe can be placed inside a medical needle [35–38], application of the technique described here is enabling for such miniaturised in-vivo Raman spectroscopy.

Here, a time-resolved SPAD line array with 512 pixels operating in a time-correlated single photon counting (TCSPC) modality, configured as a spectrometer [39] with the capability to measure up to 194 million events/s is used to detect the backscattered Raman signal from the sample. Many independent detector pixels measure the Raman wavelengths simultaneously which increases the count rate and therefore decreases the measurement time, compared to scanning systems, for practical applications. While using a SPAD array introduces an additional challenge of pixel timing variation which is not the case for a scanning system, this has been overcome thoroughly as describe in [39]. We now show how this can be exploited to achieve better signal enhancement, including single fibre probe demonstration for the first time where background noise generated in the fibre core can be separated from the sample.

## 2. Methods and materials

### 2.1. Experimental setup

**Fig. 1.**
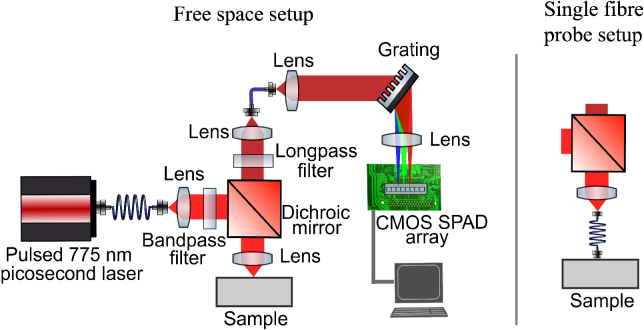
Left: Experimental setup used for free space time-resolved Raman spectroscopy using a CMOS SPAD array. A pulsed laser light is directed onto the sample, the backscattered Raman and fluorescence signal is returned and delivered to the time-resolved spectrometer. Right: Fibre probe spectroscopy is performed by placing a 1 m multimode fibre (M42L01, Thorlabs) with 0.22 NA and 50 μ m core diameter before the sample.

The experimental setup is shown in Fig. 1. A 775 nm pulsed laser (VisIR-775, 70 ps FWHM, PicoQuant) is used as biological fluorescence is less significant at this wavelength than for visible light illumination, while Raman scattering is also reduced, this is a compromise for visibility. The laser beam is collimated onto a bandpass filter (FF01-781/23-25, 781 nm CWL, 31 nm FWHM, Semrock), directed by a dichroic mirror (DMLP805R, Thorlabs) and focused onto the sample. Laser power at the sample is up to the order of 60 mW. The backscattered light from the sample is collected by the same lens and transmitted through the dichroic mirror. The Rayleigh scattering is blocked by a longpass filter (FELH0800, Thorlabs), allowing the weaker backscattered Raman to be directed through an optical fibre (M42L02, Thorlabs) onto a spectrometer composed of a collimating lens (AC254-030-B-ML, Thorlabs) with a 30.0 mm focal length, a transmission grating (1624 grooves/mm, Wasatch) for dispersion, and a focusing lens (AC508-075-B-ML, Thorlabs) with a 75.0 mm focal length. The Raman and fluorescence signal is dispersed onto a time-resolved in-house 512-pixel CMOS SPAD line sensor, sensitive over visible and near infrared wavelengths [40], and described in detail elsewhere [41]. In this configuration the spectral sampling interval of the spectrometer is 0.15 nm per pixel.

Single fibre probe spectroscopy is performed by using the setup shown on the right in Fig. 1. Here, a 1 m multimode fibre (M42L01, Thorlabs) with 0.22 NA and 50 μ m core diameter is placed before the sample. The multimode fibre is used to deliver illumination and collect spectra at 1 m distance and the bare fibre tip is in contact with the sample with the laser power at the sample approximately 30 mW.

In TCSPC mode, each of the line sensor’s 512 pixels records a histogram of photon arrival times with respect to the laser’s synchronisation signal. By combining the histograms, a multi-dimensional dataset is obtained which represents the intensities of Raman and fluorescence scattering as functions of wavelength and photon arrival time. Here the exposure time of all the measurements was 30 s. To avoid pile-up and saturation effects, count rates should be within the single-photon regime which is defined as 1-5% of the laser repetition rate. Here, the maximum detected count rate (6000 counts over 30 s with a 40 MHz laser repetition rate) is $5\times 10^{-6}$ photons per pulse per pixel. The timing resolution of the detector is 50 ps and the jitter of the detector is ∼ 150 ps full width half maximum (FWHM). A time-window of 200 ps (4 time-bins) is chosen to measure Raman scattering as this is larger than the jitter. The repetition rate of the laser was set to 40 MHz which corresponds to a 25 ns measurement window. This defines the maximum allowable photon arrival time for measurements involving optical fibres. For example, a 25 ns window limits the effective fibre length to approximately 5 m (note round trip) before a signal wraps-around and an overlap from subsequent pulses occurs. In our system the total optical path length is within this limit.

### 2.2. Data analysis

After obtaining data from a TCSPC measurement, the data are processed to reduce noise in the signal and correct for variation in detector timing by using methods described in [39]. In summary, the dark counts are calculated within each measurement, are approximately constant in time and subtracted pixel wise. Noisy pixels are removed from the data set, resulting in the removal of approximately 75 out of 512 pixels (∼ 15%). These pixels are randomly distributed and have minimal effect on the reconstructed spectra. Due to multiple data points recorded within each Raman peak (∼ 1 nm FWHM) relative to the system’s spectral resolution (∼ 0.45 nm), peaks remain well observed. The variation in the timing of the detectors is corrected for by re-aligning and interpolating the data. As high timing accuracy is needed to separate the Raman signal from background signals (fluorescence and fibre background), this step allows for short time-windows to measure Raman scattering and maximise the SNR in the final result. The photon counts are summed together across 3 pixels, resulting in 0.45 nm between each resulting data point, to increase the SNR and mitigate pixel to pixel variation. Lastly, the Raman spectra of the sample are obtained by summing four time-bins, each with a width of 50 ps, resulting in a total time-window of 200 ps. This time-window was chosen as it provides high fluorescence rejection [26,27,29].

## 3. Results and discussion

### 3.1. Separation of Raman and fluorescence

**Fig. 2.**
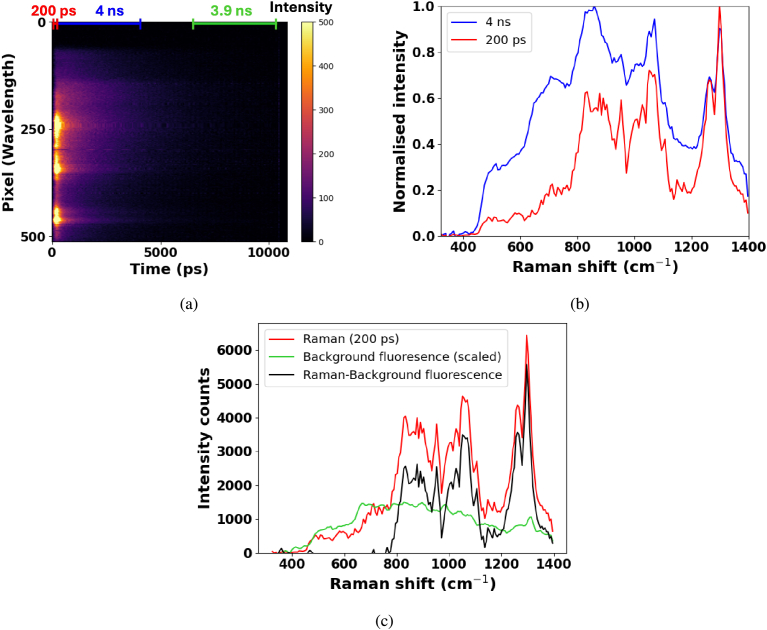
30 s time-resolved Raman spectroscopy measurement of olive oil. a) The intensity of photon counts in the TCSPC measurement is separated spectrally to detector pixels to resolve the Raman peaks of the sample. The time-bins provide temporal information about the origin of the scattering (Raman or fluorescence). b) Spectra obtained from different time-windows from the TCSPC measurement in a). The red curve represents the Raman scattering obtained in a time-window of 200 ps. The blue curve corresponds to a time-window of 4 ns. c) Removal of background fluorescent present in the 200 ps time-window spectra (red). The shape of the fluorescent background (green) is obtained from later time-bins where the Raman signal is no longer present. Subtracting the fluorescence background from the 200 ps time-window spectra results in a fluorescence free Raman spectra shown in black.

**Fig. 3.**
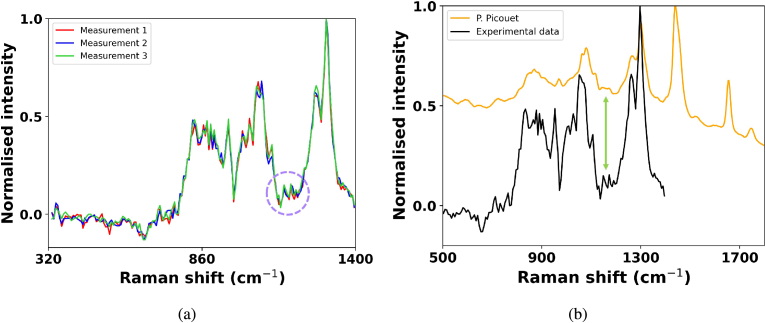
a) Raman spectra of olive oil repeated three times obtained from a time-window of 200 ps and an exposure time of 30 seconds. b) Comparison of the Raman spectra of olive oil (black) using a time-resolved spectrometer and the Raman spectra from P. Picouet (orange) [42]. Raman peaks match up to those in literature, but with increased visibility.

The data obtained from a TCSPC measurement, using the experimental setup in Fig. 1, results in a measurement as shown in Fig. 2(a). Here, olive oil is used to demonstrate the separation of fluorescence and Raman signals. The backscattered Raman signal appears at earlier time-bins, as seen in Fig. 2(a), and the fluorescent background appears at later time-bins. This is due to Raman scattering occurring instantaneously whilst fluorescence is emitted with a characteristic time delay [19]. The red curve in Fig. 2(b) corresponds to the spectra obtained from the photons arriving in a time-window of 200 ps. As this time-window is increased to 4 ns (Fig. 2(b), blue curve), more fluorescence is introduced into the Raman spectra, masking less intense Raman peaks and decreasing the SNR. This can be compared to the green curve of Fig. 2(c) which shows the shape of the fluorescence spectra, estimated by selecting a time-window later in the time-trace. Here, the time-window was selected 5 ns later than the Raman signal to minimise any residual Raman signal that might be in the extended jitter tail of the detectors. This background can be subtracted from the Raman spectra to obtain the background free Raman spectra shown by the curve in black (Fig. 2(c)). Using this technique to remove fluorescence in the spectra avoids introducing errors caused by baseline subtraction techniques as the form of the background is measured rather than assumed. However, the technique is vulnerable to correct scaling of the background fluorescence and may not accurately reflect the full shape of the fluorescent background. For example, in Fig. 3(a), the negative values in the 320-700 cm^− 1^ range are caused by an overestimation of the background fluorescence as scaling is performed at the lower end of the region of interest at 770 cm^− 1^. While fluorescence lifetimes were observed to be nominally the same across the spectra range, slight variation may cause over estimation of fluorescence at lower Raman shifts and skew the subtracted spectra. Despite this limitation, from a single TCSPC measurement the visibility of small and fine features has increased in the resulting spectra after time-gating, while also allowing the fluorescence background to be observed for baseline subtraction.

By measuring the full TCSPC trace, additional fluorescence lifetime information about the sample can also be acquired. Figure 2(c) is an example of a measurement where the Raman spectra and the fluorescence lifetime (approximately 2 ns) of the sample was obtained from a single measurement with a 30 s exposure time. Detecting fluorescence and Raman during the same measurement could further reduce measurement times for applications which require both information from a sample as multiple measurements do not need to be performed.

### 3.2. Pixel sensitivity and repeatability

Figure 3(a) demonstrates that the Raman peaks observed for olive oil are consistent across multiple measurements, indicating that the peaks are repeatable and due to Raman scattering. To ensure the smaller peaks, highlighted by the purple circle at 1160 cm^− 1^, were not due to random noise, shot noise or system fluctuations, the measurements were repeated three times and compared to peaks present in reference data highlighted by the green arrow in Fig. 3(b). If the peaks were due to pixel sensitivity variation these features would also appear in the fluorescence spectra shown by the green curve in Fig. 2(c) and in the full data shown by the blue curve in Fig. 2(b). However, these features only become apparent with the improved visibility of Raman from the sample offered by this technique. Computational baselining of the full data would not offer this improvement as the features are masked in the shot noise. The sawtooth appearance of certain peaks in this figure is due to sampling of the signal as discussed in section 3.3. Correction of timing characteristics of the individual pixels was critical to achieving this result [39]. If this was not properly corrected, systematic noise qualitatively similar to the highlighted Raman features could be present, however, this is not observed where it would be present on time-gated measurements of smooth spectra such as the tip of the fibre optic discussed in Section 3.3. Comparison to reference data from the literature (Fig. 3(b)) with good correlation gives further confidence in the origin of these Raman features.

Figure 3(b) compares the baselined Raman spectra of olive oil from this study with the spectra obtained by P. Picouet [42] using a QEPro (Ocean Insight, USA) with a 785 nm laser operating at 400 mW. The orange spectra, obtained by Picouet, has an overall exposure time of 10 s and a resolution of 11 cm^− 1^, whereas the spectra from this work has a comparable resolution and was obtained at a lower laser power (∼ 60 mW). The removal of fluorescence improves the SNR, allowing for better visibility of specific spectral peaks, for example the peaks at 1160 cm^− 1^ highlighted by the green arrow. The improved visibility of the spectral peaks allows for differentiation of similar samples, or samples that have been adulterated.

**Fig. 4.**
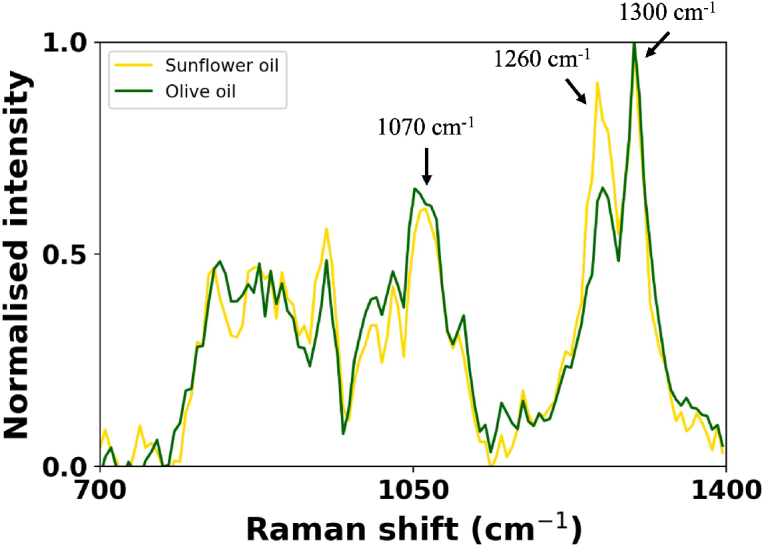
Comparison of the Raman spectra of olive oil (green) and sunflower oil (yellow). Time-window 200 ps, total exposure 30 s.

Figure 4 shows a comparison between the Raman spectra of olive oil (green curve) and sunflower oil (yellow curve) obtained using the same experimental setup and parameters as Fig. 2. Many peaks are in common, however, peak amplitude varies with molecular composition of the two samples. The peak visible at 1070 cm^− 1^ is due to the C–C stretching, the peak at 1260 cm^− 1^ corresponds to the =C–H deformation and the peak visible at 1300 cm^− 1^ is due to the C–H bending in methylene (CH_2_) [43]. This further confirms these features are indeed sample dependent Raman features previously obscured by fluorescence and not system artefacts.

### 3.3. Single fibre probe spectroscopy

**Fig. 5.**
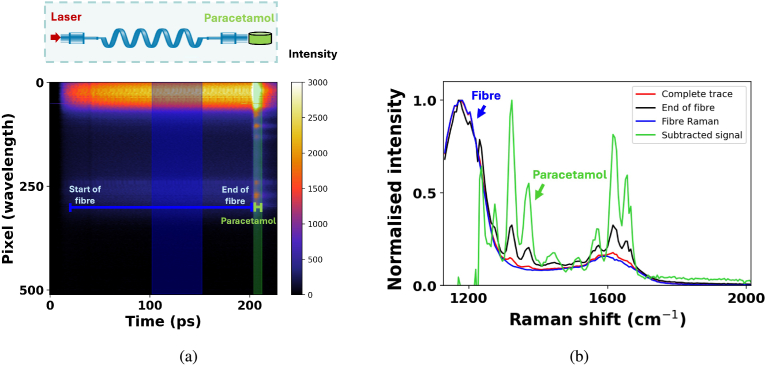
TCSPC measurement of a single fibre probe setup with a solid 500 mg paracetamol placed at the distal end of the fibre. The laser power at the sample is ∼ 30 mW. b) Spectra obtained from different time-windows in (a). The red curve shows the spectra obtained from the entire time-trace. The black curve shows the spectra obtained from the time-window at the end of the fibre, where peaks from the fibre and the paracetamol can be seen. The blue curve corresponds to the Raman spectra from the optical fibre and the green curve is a subtraction of the red and green curve to obtain the Raman spectra of the paracetamol.

Figure 5(a) shows the entire TCSPC trace from a single fibre probe measurement. As the backscattered Raman photons from the optical fibre and the paracetamol have travelled different path lengths they will arrive at the detector at different times. The difference in time-of-flight is used to separate Raman signal from the optical fibre and the paracetamol in the time-domain. In Fig. 5(a) the constant Raman scattering along the length of the 1 m optical fibre probe is observed from time-bins 10 to 205. At the end of the fibre, from time-bin 205 to 210, the reflection of the forward scattered fibre Raman caused by the glass-air interface in addition to the Raman signal from the paracetamol can be seen.

The red curve in Fig. 5(b) shows the Raman spectra of the entire trace, where the peaks due to Raman scattering generated inside the optical fibre core are visible at 1200 cm^− 1^ and 1600 cm^− 1^. The peak present at 1600 cm^− 1^ is not as strong as the characteristic peaks more commonly observed in glass at 500 cm^− 1^ and 1200 cm^− 1^ [44,45], however, it has been observed in previous work [32,33]. The broad nature of the peak suggests it is due to an amorphous material, this could be the acrylate coating of the fibre or from contamination of the glass fibre during manufacturing (such as carbon) and not necessarily intrinsic to the glass. Figure 5 provides evidence that this Raman feature is generated along the length of the fibre as it occurs at earlier time-bins than the signal from the Raman sample and is therefore intrinsic to this type of optical fibre. The smaller peaks present at 1325 cm^− 1^ and 1615 cm^− 1^ are characteristic peaks of paracetamol [46]. By analysing the time-bins at the end of the fibre, shown by the black curve in Fig. 5(b), the Raman peaks from the paracetamol become more visible. The presence of peaks from the Raman generated in the optical fibre are still visible due to forward scattering Raman signal reflecting off the fibre tip and paracetamol sample and returning simultaneously to the detector. To remove the Raman generated in the fibre, its spectrum shown by the blue curve in Fig. 5(b) is subtracted from the spectrum obtained at the end of the fibre. The resulting spectra, displayed in green, is the Raman spectra of paracetamol alone.

**Fig. 6.**
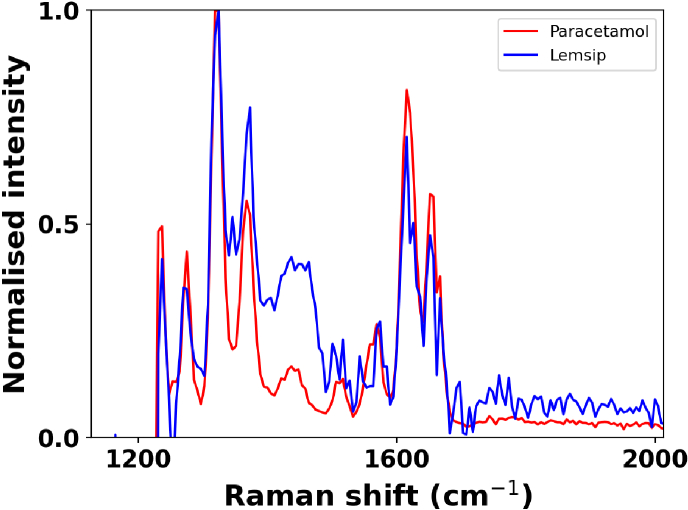
Comparison of the Raman spectra of paracetamol (red) and Lemsip Max (blue) obtained using a single fibre probe with a 30 s exposure time and a 200 ps window to remove fluorescence.

Figure 6 shows the ability of the single fibre probe to differentiate a pure sample (paracetamol) and a mixture (Lemsip Max). The paracetamol is a 1000 mg solid tablet whereas the Lemsip Max is a powder containing 1000 mg of paracetamol together with additional active and excipient ingredients such as phenylephrine hydrochloride 12.2 mg [47]. The difference in the Raman spectra is due to the presence of other ingredients in the Lemsip compared to the pure paracetamol. Paracetamol has a well-studied Raman response and aids in characterising the spectral resolution of this system. Data presented here has a 0.45 nm spectral sampling interval, referring to the spacing between data points (nm/pixel) equating to ∼ 7 cm^− 1^. A measure of the true optical resolution depends on optical bandwidth, grating size, detector pixel size and data sampling. In Fig. 5 and 6 the sharpest peak, at 1275 cm^− 1^, has a width (FWHM given by a Gaussian fit) of 12.7 cm^− 1^. This peak is only represented by a few data points and the observed sharpness may be limited by sampling. True spectral resolution is either limited by the sampling interval or is comparable to the optical system resolution. Here, the measured resolution is comparable to that of reference data [42] shown in Fig. 3(b). The spectral sampling interval is a limitation of this work, causing spectra to be under sampled as noted. This is not normally the limitation in scanning or higher pixel number spectrometer configurations. In the future, this limitation could be overcome by using SPAD arrays with more pixels or by studying a smaller spectral range.

## 4. Conclusion

Here, time-resolved techniques were used to separate sample Raman spectra from unwanted background signals such as sample fluorescence and Raman generated in the single fibre probe. During a 30 s measurement, the Raman spectra of a sample is obtained by selecting the photons arriving in a time-window of 200 ps. Any background fluorescence which appears in this time-window can be estimated and then subtracted from the Raman spectra by selecting a time-window later in the time-trace where no Raman scattering occurs. Measurement time is reduced here by dispersing the different Raman wavelengths across a SPAD array which can simultaneously detect the photons across the entire spectral range compared to individual detectors which scan each wavelength individually. This technique was also used in a single fibre probe setup to separate the Raman signal from the sample at the distal end of the fibre and the optical fibre itself. Consequently, fibre probes can be designed to be smaller, with the limiting factor being the optical fibre rather than the lenses at the probe tip, as is commonly the case. The combination of practical measurement time, enhanced Raman visibility, and implementation with a single unmodified optical fibre is enabling for translation and impact in healthcare applications as a miniature probe.

## Data Availability

Data underlying the results presented in this paper are available in [48].
